# Preoperative AI-Driven Fluorescence Diagnosis of Non-Melanoma Skin Cancer

**DOI:** 10.3390/diagnostics12010072

**Published:** 2021-12-29

**Authors:** Victoriya Andreeva, Evgeniia Aksamentova, Andrey Muhachev, Alexey Solovey, Igor Litvinov, Alexey Gusarov, Natalia N. Shevtsova, Dmitry Kushkin, Karina Litvinova

**Affiliations:** 1 Moscow Regional Research and Clinical Institute (MONIKI), 129110 Moscow, Russia; viktoriaa@yandex.ru (V.A.); dr.aksamentova@gmail.com (E.A.); natniksh@gmail.com (N.N.S.); 2 Dermclinic LLC, Bannyy Pereulok 2c2, 129110 Moscow, Russia; info@dermclinic.ru; 3 Deep Smart Light Ltd., 7 Bell Yard, London WC2A 2JR, UK; andreymja@gmail.com (A.M.); soloveybird@gmail.com (A.S.); info@deepsmartlight.com (I.L.); gusarov.alex3@yandex.ru (A.G.); 4 The Advanced Educational Scientific Center, Kolmogorov’s Boarding School, Moscow State University (AESC MSU), 121357 Moscow, Russia; 5 Moscow Power Engineering Institute, National Research University, 111250 Moscow, Russia; 6 Department of Bioengineering, Imperial College London, South Kensington, London SW7 2BT, UK; 7 Aston Medical School, College of Health & Life Sciences, Aston University, Aston Triangle, Birmingham B4 7ET, UK

**Keywords:** non-melanoma skin cancer, basal cell carcinoma, fluorescence diagnostics, artificial intelligence, neural network, deep learning, dense neural network

## Abstract

The diagnosis and treatment of non-melanoma skin cancer remain urgent problems. Histological examination of biopsy material—the gold standard of diagnosis—is an invasive procedure that requires a certain amount of time to perform. The ability to detect abnormal cells using fluorescence spectroscopy (FS) has been shown in many studies. This technique is rapidly expanding due to its safety, relative cost-effectiveness, and efficiency. However, skin lesion FS-based diagnosis is challenging due to a number of single overlapping spectra emitted by fluorescent molecules, making it difficult to distinguish changes in the overall spectrum and the molecular basis for it. We applied deep learning (DL) algorithms to quantitatively assess the ability of FS to differentiate between pathologies and normal skin. A total of 137 patients with various forms of primary and recurrent basal cell carcinoma (BCC) were observed by a multispectral laser-based device with a built-in neural network (NN) “DSL-1”. We measured the fluorescence spectra of suspected non-melanoma skin cancers and compared them with “normal” skin spectra. These spectra were input into DL algorithms to determine whether the skin is normal, pigmented normal, benign, or BCC. The preoperative differential AI-driven fluorescence diagnosis method correctly predicted the BCC lesions. We obtained an average sensitivity of 62% and average specificity of 83% in our experiments. Thus, the presented “DSL-1” diagnostic device can be a viable tool for the real-time diagnosis and guidance of non-melanoma skin cancer resection.

## 1. Introduction

Keratinocyte carcinomas (KC) or non-melanoma skin cancer is a group of malignant skin neoplasms (MSN), which includes basal cell carcinoma (BCC), which occupy from 75.0% to 97.0% of all malignant epithelial neoplasms of the skin, squamous cell carcinoma (SCC), which accounts for 5.0% up to 15.0%. Rare skin appendage carcinomas (sebaceous and sweat glands, hair follicles) constitute less than 1.0% of all types of KC [1,2,3]. In the structure of the incidence of malignant diseases, KC in recent decades occupies from first to third place in most countries of the world [4]. Malvehy et al. note that KC accounts for 80.0% and 20.0% of all types of MSN [5]. BCC is the most common skin neoplasia [6]. The incidence rates of BCC differ in regions, reaching extremely high in countries with hot climates. In Australia, in 2012, the incidence was 336.0 per 100,000 of the population in men and 251.0 per 100,000 of the population in women [7,8,9,10]. According to the Skin Cancer Foundation, the incidence of KC in the United States each year exceeds the total rate of malignant tumors of the breast, prostate, lung, and large intestine [11]. KC can cause significant local damage and can cause metastasis if left untreated. Thus, untreated and neglected BCC leads to infiltrative changes. Due to the infiltration of deeper tissues and blurred borders, the treatment of neglected BCC is quite tricky [1,6,11,12]. Leaving even a small focal infiltration will result in the recurrence of the disease. However, there is no tendency for metastases to occur in BCC, whereas SCC is associated with metastatic risk and often turns out to be more aggressive and more challenging to treat [1,2,3,6,11,12]. The gold standard for diagnosis of skin lesions is biopsy and subsequent histopathologic correlation. The procedure is both invasive and time consuming, which severely limits its use, for example, intraoperatively. Moreover, the biopsy requires several highly qualified specialists to collect tissue samples for histological examination and microscopic evaluation. Thus, the human factor could also be attributed to the disadvantages. All of the above suggests the need for additional and improved evaluation methods of patients with KC at the preoperative stage.

Skin lesion screening is one solution for the early detection of KC. Nowadays, lasers have become an essential tool for creating the next generation of innovative diagnostic technologies. It offers new ways to prevent, diagnose, and monitor health complications. The laser-based technique takes much less time to acquire high-quality spectral signals [13]. Fluorescence spectroscopy (FS) is an excellent tool for non-invasively obtaining valuable biochemical information related to the metabolic properties and structural components of the extracellular matrix in tissue [13,14]. This technique is rapidly expanding due to its safety, relative cost-effectiveness, and efficiency [15,16,17]. Considering that FS was already shown as an effective tool for skin cancer detection, there is still a significant challenge with the technique, as it generates a lot of spectral and complex data related to the great variety of benign and malignant forms of skin pathologies. Particularly, BCC lesions have more than 15 subtypes, SCC lesions have about ten different subtypes, and all of them have a variety of benign and dysplastic forms, which differ by morphology, appearance, and metabolic statement, including by their optical properties, on the different stages on the lesion growth [1,6,11].

Artificial intelligence (AI) and machine learning (ML) techniques have been widely applied in the detection of skin cancer [18]. Deep Learning (DL) is the subset of ML to mimic the human brain’s ability for data processing. It is considered the most difficult subcategory of training related to the algorithms of artificial NN. DL is widely used in diagnostics and currently has achieved diagnostic efficacy in classifying skin cancers with a level of competence comparable to dermatologists [18,19,20,21,22,23,24]. The most common inputs for deep NN are visual images from photos of the site or grayscale images of ultrasound inspection. The only 3D input that could be treated by NN that will be real 3D is the magnetic resonance imaging (MRI) or positron emission tomography (PET) images where each voxel (3D pixel) is segmented into tissue type: “normal” or “not-normal” (pathological). Historically, the first NN used for segmentation worked with a similar problem: segmentation of biomedical images [25]. The classification and segmentation problem that can be solved by DL and cannot be solved by dermatologists or clinical radiologists is the problem when perception field cannot be perceived by human sense organs: vision, hearing, etc. Even researching the invisible in vision wavelengths picture (X-Ray, MRI, PET) is being transformed into 2D grayscale that could be easily perceived by clinical imaging professionals [20,21,22].

An input tensor for the DL may contain the broader range of wavelengths that could be visually perceived and the broader dimensions, such as extinction wavelength and extinction intensity. The picture of high dimensions could be perceived by humans, usually in the projection form, when the 2D projections are provided. There are no limitations both in the tensor width and in the tensor dimensions for the DL. The challenge for this multidimensional input is to ensure proper and accurate segmentation. At the moment, this can be done by only performing a histological examination.

**Figure 1:**
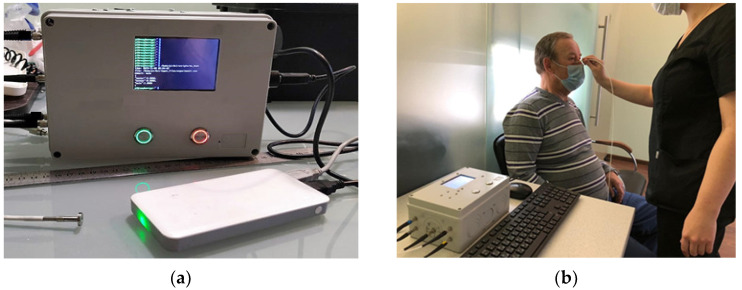
Experimental setup of the DSL-1, where: (**a**) DSL-1 prototyped device; (**b**) Optical bundle of the DSL-1 is placed in gentle contact with the under eyes skin lesion for fluorescence spectra recording.

DSL-1 is a portable, non-invasive, laser-based skin cancer diagnostic device that detects biochemical information of the skin using FS backed with a DL-based diagnostic algorithm (Figure 1a). The primary objective of this work was to collect fluorescence spectra of skin, benign lesions, BCC, and SCC to construct the database, validate the classifying algorithm, and accurately predict the probability of BCC for individuals and patients. From the diagnostic point of view, the described situation and setup are precisely related to those multiple perceptions that would be difficult for humans to perceive but can easily be perceived by DL. The ability of the diagnostic device to distinguish between benign and malignant abnormalities was the main component of accuracy in a risk assessment process. This task required improving vision both in terms of wavelengths and the various spectral experimental installations of FS.

## 2. Materials and Methods

### 2.1. Patients

The independent Ethics Committee of the Dermatology Clinic “Dermclinic LLC” approved this study (protocol No. 3 on 8 September 2020). All patients provided informed consent before the examination.

The study included 137 patients (74 males, 62 females) with various forms of primary and recurrent BCCs (median age—56 years, range 30 to 70 years) and 287 (149 males, 138 females) healthy volunteers (median age—51 years). The percentage of patients with more than one BCC lesion was 34%. All of the BCC lesions were confirmed histopathologically.

### 2.2. Fluorescence Measurements and Data Preparation

The fluorescence spectra were recorded from BCC lesions and the surrounding skin before biopsy and treatment. Five measurements were taken from different spots within the lesion area. Another five measurements were taken from different spots of the surrounding normal-appearing skin 2–4 cm away from the visible border of the tumor.

The fluorescence spectra were measured with a laser non-invasive diagnostic prototype DSL-1 (Deep Smart Light Ltd., London, UK) (Figure 1). The DSL-1 consists of the light collection module and the spectral analysis module. The light collection module comprises 4 different lasers (340–850 nm wavelengths range) and is equipped with a 2 mm optical bundle. The bundle houses a combination of multiple optical fibers, each employed to either deliver or detect light. The output power at the bundle-end is around 3 mW for each light source, and the approximate diagnostic volume within tissue is about 2–3 mm^3^. When the lasers were irradiated, the emission spectra of tissue were automatically generated from the microspectrometer inside the device and simultaneously displayed on the DSL-1 monitor and stored in an in-built microcomputer with NN. This NN architecture has been determined during our previous preclinical ex vivo and in vivo studies [26]. The trained NN was uploaded into the device memory and allowed the automated classification of the spectra in real time. For training purposes, the initial set of spectra was split into two parts: training and validation. Then, the trained DL algorithm determined whether the skin lesion was normal (intact, benign) or BCC based on the unique multispectral signature.

### 2.3. Dataset Preparation, Experimental Setup, the Architecture of the NN and Training Algorithm

The fundamental purpose of the DL solution was to make a decision on whether these spectra correspond to cancer or normal tissue by using 4 fluorescence spectra. The ML problem that is used to solve this problem is binary classifier (BC). As a result of the high dimensions of the input data (88 × 4), the dense neural network (DNN) solution was selected. Three dense layers (64, 64, 128 neurons) address the problem to be sufficient for reproducing the non-linear functional dependencies, the sequence of activation functions—Rectified Linear Unit, Sigmoid, and Hyperbolic Tangent—were selected to emulate the series of physical signal processing: Filtering (ReLU), Compiling Non-Linear Signals (Sigmoid), and Making Decision (Tanh). The Dropout Layers were put after second and third dense to prevent overfitting. Due to the lack of the possibility of input augmentation, the fit-generator method was not used. The classical converging method of stochastic gradient decay with classifier-related improvements of momentum and step decay was used.

**Figure 2:**
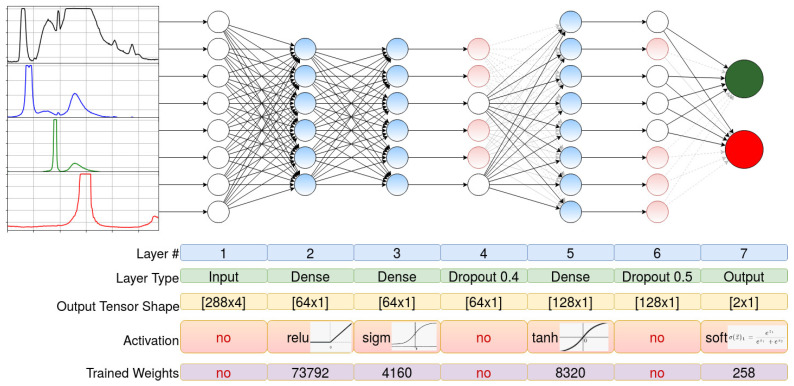
The architecture, loss function, and fitting parameters of the DSL-1 DNN.

For BC, researchers widely use one output neuron and the corresponding loss of binary cross-entropy. Due to the physical and clinical meaning of the decision, the classifier could be naturally extended to a categorical classifier with a significant number of decisions. It was the main reason for using two output neurons. Such output architecture allowed us to solve two problems: firstly, the loss function became categorical cross-entropy, which allowed DNN to learn more steadily, and secondly, it doubled the number of weights in front of the last layer, which made it possible to increase the generalizing ability. The DNN architecture, loss function, and fitting parameters are given below and represented in Figure 2.

DNN Layers Architecture:

1. Input (288 × 4)
2. Dense (64, activation = relu)
3. Dense (64, activation = sigmoid)
4. Dropout (0.4)
5. Dense (128, activation = tanh)
6. Dropout (0.5)
7. Output Dense (2, activation = softmax).

Training Prerequisites:

1. loss: categorical_crossentropy
2. learning_rate: 1 × 10^−6^
3. learning rate decay: 1 × 10^−6^
4. momentum: 0.9
5. nesterov: True
6. epochs: 15,000
7. batch size: 32

early stopping:

- monitor: val_loss
- patience: 5000 epochs.

checkpoints:

- es: early stopping
- mc: Model Checkpoint, monitor: val_loss, save_best
- validation_data: X_test, Y_test.

There were 286 cases: 149 skin or “non-cancer” and 137 cancer cases. Each case referred to a single patient and a single type of tissue/lesion. Bearing in mind the variation in the sizes of lesions, sometimes, the case had several spectra (the potential skin cancer site was measured several times from different angles). Therefore, the entire number of “train + test + validation” samples was 804. To make the experimental setup more clear, we never used the same case in the test, train, or validation set.

The pseudocode of the experiment was as follows:

- Run the 50 independent experiments of:
- Split the 286 cases randomly into 3 parts: train (229), test (29), and validation (28);
- Run training on the train set, using loss on the test set for early stopping;
- Evaluate sensitivity and specificity on the validation set by using the “best-by-accuracy-on-test-set” model saved in the “mc-checkpoint”.

## 3. Results

**Figure 3:**
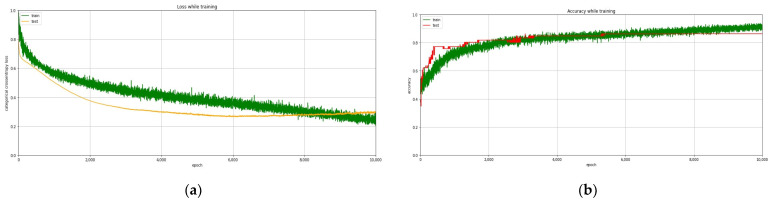
Training curves for BC: (**a**) Training process: loss; (**b**) Training process: accuracy.

An important part of the training process involved matching the skin tissue fluorescence spectra of patients with primary and recurrent BCCs and further refining the DL algorithms and confirming the appropriate spectra for “normal skin” and “cancer”. We implemented DNN, which was trained to differentiate healthy and cancerous lesions. For the training of the DSL-1 DL algorithms, 692 spectra were provided: 418 “cancer” spectra and 274 “normal” skin spectra. For validation, the separate asset of patients was used. There were 112 spectra in the validation set: 68 “cancer” spectra and 44 “normal skin” spectra. The training curves for BC are represented in Figure 3.

At the end of the DNN, two output neurons were presented (Figure 2). The outputs of these neurons have been transformed using the softmax function. In this way, the output values of the neurons could be treated as “probability”. If neuron “2” had a value ≥ 0.5, it was classified as “cancer”. For example, if multiple spectra measurements (N) have been made for a patient and more than N/2 spectra have been assigned the correct class, it is assumed that all spectral data for that patient are correctly classified.

To ensure stable numbers, we provided 50 cross-validation experiments. Each experiment had separate sets of patients for training, testing, and validation. The DNN was trained on a training set, the testing set was used for early stopping. The testing set was not used in the training process, only to assess the values of sensitivity and specificity. The statistics of the sensitivity and specificity values on the validation set are shown in Table 1 below.

## 4. Discussion

According to the literature data, FS is a rapidly emerging non-invasive technique that can detect structural and chemical alteration in skin lesions [27]. Skin FS can give information on the presence and quantity of a wide range of diagnostically relevant molecules, including amino acids, lipids, metabolic cofactors, and heme precursors. Using laser sources, molecules of interest can be specifically targeted and studied in tissues based on the intrinsic excitation and emission properties. In previous human studies, we demonstrated that collagen-related fluorescence intensity decreases in the BCC compared to the normal intact tissue [28,29]. Other studies have also reported reduced fluorescence intensities at the spectral emission peaks of NADH and FAD for BCC lesions compared to healthy tissue [15,16,17,30,31,32]. Despite noticing differences in the fluorescence intensity profiles, numerous authors reported low values for accuracy when differentiating between healthy and BCC tissues [33,34]. Additionally, there is still a significant challenge with implementing the FS technique into the routine clinical setting.

Since the FS technique generates plenty of images and large quantities of data, which should be coded and displayed in a manner that specialists can easily extract relevant information, AI algorithms can be trained to classify the skin lesions without being overwhelmed by an excess of data. This trial presents the results obtained with the multiple wavelength excitation of the endogenous fluorescence of benign and malignant skin lesions using a fluorescence-based prototyped diagnostic device with integrated NN for skin cancer classification. Spectral data were detected of lesions and the surrounding normal skin fluorescence using different excitation wavelengths in the spectral range 340–850 nm. We already have investigated more than 230 clinical cases to receive the spectral properties of BCC and benign cutaneous lesions. The NN architecture, loaded into DSL-1 and described in this study, had to meet and satisfy the problem setup, since it is a classification problem {spectra} ≥ {solution class}. Here, we demonstrate the effectiveness of DNN, which was trained end-to-end from fluorescence spectra directly, using only pixels and disease labels as inputs in the classification of skin lesions. These results also highlight AI-aided fluorescence diagnosis ability to determine the multispectral molecular signature and make a decision on whether these spectra correspond to cancer or normal tissue. For BC problem (cancer/not cancer), the sensitivity/specificity values are the most related metrics to discuss the performance of the method. For 286 cases and 692 different spectral sets, we ensured the cross-validation technique. All cases were split into three groups: training, testing, and validation. The training set was used for training, and the testing set was used for early stopping whilst training. In this way, we deliberately reduced the training set size to gain accurate numbers. In our experiments, we obtained an average sensitivity of 62% and an average specificity of 83%. These values of sensitivity and specificity are quite similar to the study reported by A. Dascalu and E. David [35]. The authors proposed a computer-assisted diagnostics system that improves the diagnostic accuracy of a low-quality dermoscope. They have analyzed 73 patients (biopsies) with skin cancer and achieved an accuracy of detection of 81.4% (sensitivity 89.5%; specificity 57.8%).

**Figure 4:**
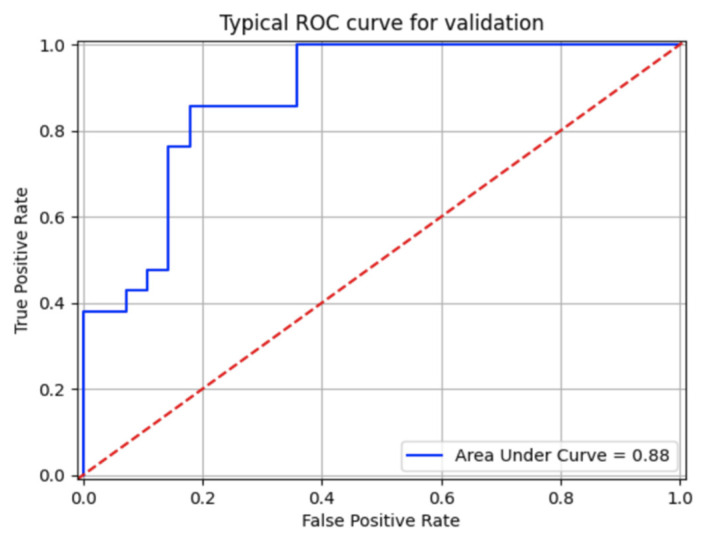
An example of one of the ROC curves on the validation set: 30 spectral sets of the validation set (seven patients, 10 cases). The area under the curve (AUC) is 0.88.

If we use all the patients for the training and testing (not for the validation), the quality of the method is definitely increased. As an example, Figure 4 shows a typical ROC curve for one of 50 cross-validated experiments.

The inputs of the proposed DNN were intensities of fluorescent spectra on different wavelengths on certain extinctions, so all the inputs were of the “same-nature”. Convolutional NN is a common solution to the NN architecture for “same-nature” inputs [18,35,36,37,38,39,40,41,42]. Convolutional layers have two advantages over fully connected layers: a limited field of view and a significantly reduced number of training parameters that prevent overfitting [36,37]. However, it should be noted that the architecture of our solution was based on fully connected layers, not convolutional. We had a strong motivation for that, which comes from physics and understanding the nature of the fluorescence spectra. Intensities on some particular wavelengths are not the same as those for the other wavelengths—they represent the concentration and the corresponding fluorescence of certain substances. Our method is an indirect probe of the concentration of specific metabolites and substances, and their spectral “signature” cannot be translated onto some wavelength range. Therefore, fully connected layers were our solution for the NN applied in this study. Locally connected layers also would not work in our case, mainly because the fluorescence spectra of certain substances appear in different wavelength ranges [43]. Since the NN was built by fully connected layers, dropout layers were essential to prevent memorizing the train spectra and, as a result, to dodge the overfitting. Accordingly, the dropout levels were selected to be high enough (0.4, 0.5) for the entire decision-making system to be as robust as possible. Our DNN classification results were successfully compared and confirmed by histopathological analysis.

## 5. Conclusions

The method described in this paper shows good statistical values: a specificity of 0.83 and sensitivity of 0.62. Due to the neat cross-validation technique, no overfitting has been identified. The clinical research is currently under implementation; thus, it will become possible in future work on a large dataset from more substantial patient numbers to gain the best prediction and classification accuracy. By now, we have received a good correlation between histopathological analysis of the skin lesions and DNN classification. With the construction of the database with the spectral signatures of BCC, SCC, benign lesions, and normal tissues using DSL-1 and validation of the classifying DL algorithm, we expect to receive a target device for the detection and evaluation of skin lesion type.

## 6. Patents

The non-provisional patent application 16/798,001 “Non-invasive, multispectral-fluorescence characterization of biological tissues with machine/deep learning” was filed to the United States Patent and Trademark Office on 21 February 2020. International patent application PCT/IB2021/050844 was filed to Patent Cooperation Treaty (PCT) in WIPO member countries on 3 February 2021.

## Figures and Tables

**Table 1:** Sensitivity and specificity statistics.

	N	Min	Max	Mean	Median	Std	25th Perc	75th Perc
Specificity	50	0.34	1	0.83	0.85	0.17	0.75	1
Sensitivity	50	0.16	1	0.62	0.64	0.23	0.5	0.8

## Data Availability

Our GitHub repository is https://github.com/Deepsmartlight/NN.
